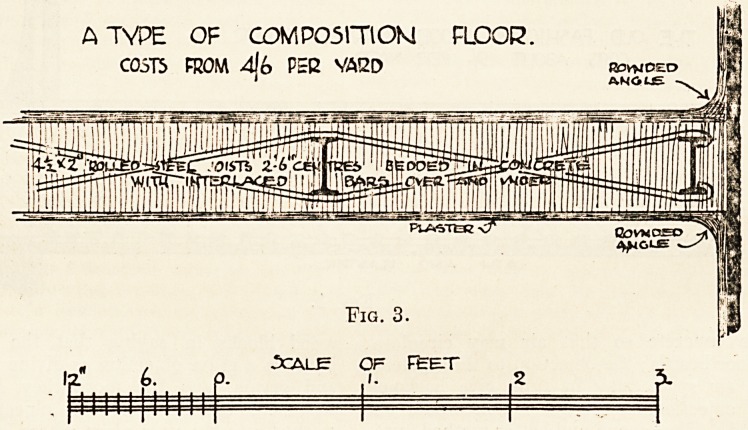# The Ward Floor

**Published:** 1911-01-28

**Authors:** 


### Hospital Architecture and Construction.

[Communications on this subject should be marked "Architecture" in the left-hand top corner of the envelope.]

### THE WARD FLOOR.

### II.—PRACTICAL DETAILS.

In the last article we confined ourselves mainly to general considerations as affecting ward floors. We will now proceed to give some practical details.

**Floor Laying.**—There are two ways of laying boarding, and in this statement we confine ourselves to the fact that modern hospitals of any pretensions have concrete and steel floors, not the old-fashioned wood joists (see fig. 1). The first method is to lay the boarding on fillets, which in their turn rest on the concrete so that air may circulate below the floor boards; the objection to this system is that the air space may in time give harbour to dirt and objectionable matter which would be better avoided. The second and better method is to lay the boarding direct on to the concrete, secured to fixing blocks bedded therein, the vital requirement in this system being that the back or underside of the boards should be coated with a preparation of pitch to avoid either wet or dry rot, depending on the amount of moisture present.

**Boards v. Wood Blocks.**—Oak blocks, usually about 9 inches by 3, are also used for flooring (fig. 2), but are held by some authorities to be undesirable, as they will not stand too much scrubbing. Teak stands water better, but probably a selected maple wood block is the best for a solid floor; it is hard and tough-grained, and will stand a great deal of wear and tear, as may be evinced from the fact that it is the most popular material for skating-rink flooring—a severe enough test in all conscience. We may, however, sum up the question of wood boards and wood blocks in saying that the multiplicity of joints is a grave objection as giving harbour to dust and germs, and the more they are polished or scrubbed, the more is the objectionable matter worked into the very place where it should not be.

Fig. 1.

Fig. 2.

**Terrazzo and its Types.**—Terrazzo, a composition of marble chips with a matrix of cement and sand is a material in demand, and even where the actual floor is of wood a surround or edging of terrazzo is often found in order to get a cavetto or rounded angle at the junction with the wall, thus avoiding corners which favour the accumulation of dust. This changing from one material to another to get the rounded angle against the walls, and in the case of stoves and radiators to get a material not affected by heat, means a multiplication of joints which, if possible, should be avoided, for butt joints have always an inclination to open. In recent years many compositions of the terrazzo type have appeared which can be used for flooring, such as mosaics, pearl inlays, and varied marbles to give different effects, and the salient advantage of this material is that it can be carried over the floors and up the walls for a certain distance, with the usual rounded angle, and when complete can present a handsome appearance; but its great objection is that it strikes very cold to the feet, is very hard, metallic, and liable to crack, and, what is still worse, it is extremely slippery unless well gritted.

**Rubber Flooring** about three-eighths of an inch in thickness and of a very hard consistency makes a very satisfactory and a comfortable floor; although laid like tiles, it is bedded in material closely akin to itself in structure, and may practically be called a jointless floor, but its present cost makes it almost prohibitive in comparison with other materials.

**Linoleum** of a good quality has frequently been used as a floor covering, more particularly on old wood floors, and has proved satisfactory; it is warm to the feet, of pleasing appearance, and has few joints; it is also less noisy than a bare floor.

Probably the best type of floor for future development, which possesses all the advantages of linoleum, is laid right on to the concrete in the same manner as terrazzo or cement, is now being used. It is to be had in various forms; some kinds have asbestos among their constituents, a material particularly lending itself to this sort of work. A careful analysis has been made of one type for the purpose of illustration :—

| | | | |
|---|---|---|---|
| Water ... ... | 33.75 | Lime ... ... ... | 5.42 |
| Carb. Acid ... ... | 5.50 | Magnesia ... ... | 30.44 |
| Sawdust ... ... | 3.42 | Mag. Chloride ... | 11.25 |
| Insoluble Residue ... | 9.03 | Sulph. Acid ... | 0.18 |
| Silica ... ... | 0.58 | Alkal. and Loss ... | 0.10 |
| Oxide of Iron .. | 0.33 | | |

The nature of this composite material is such that it can be made almost in any colour, and is applied more or less in the same manner as a cement floor (fig. 3), so that a clear field is open for any method of decorative treatment. The makers of these compositions claim that they will last, and as their nature is imperishable there is no reason to refute their claim. Care, however, should be taken when using this type of floor finish that any metals in contact are properly coated with pitch or some such preservative to counteract a tendency to corrosion caused by the acids.

**The Composite Floor.**—The cost is another of the composite's salient advantages, and recommends it for general use. As a flooring it costs slightly more than half as much as a good hard-wood floor laid complete, and, further, takes a good polish; its thickness is only about half an inch, whereas wood blocks take up an inch and a half to two inches when laid. This means a saving of an inch and a half in the height of each floor, which in a building of several stories means a saving in the initial cost not to be despised. Though rubber is the best floor for noiselessness, it has been already shown that on the score of expense it is not a practicable material; but this composite floor now under consideration seems to be less noisy than a wood floor owing to its want of resonance, and also has the advantage of being jointless. It is also damp- and germ-proof, and, what is yet of more consequence, it is quite fire-resisting. A test has been carried out by placing several samples in a Primus burner, and except for a slight discoloration on the edges, which of course would be non-existent in actuality, it has stood the test of fire surprisingly well, and even when the samples have been plunged in cold water straight from the burner no defects have appeared except that the polish on them has gone off. A further qualification of this composite material is that it can be laid either on wood or concrete, so that in wards where the existing flooring is uneven or defective it can be laid on top of it, thus giving a fresh hygienic finish. One type of this floor has been used in a grand staircase at the Brussels International Exhibition, but unfortunately no report is yet to hand on its behaviour in the recent rapid and severe fire.

FIG. 3.

## Figures and Tables

**Fig. 1. f1:**
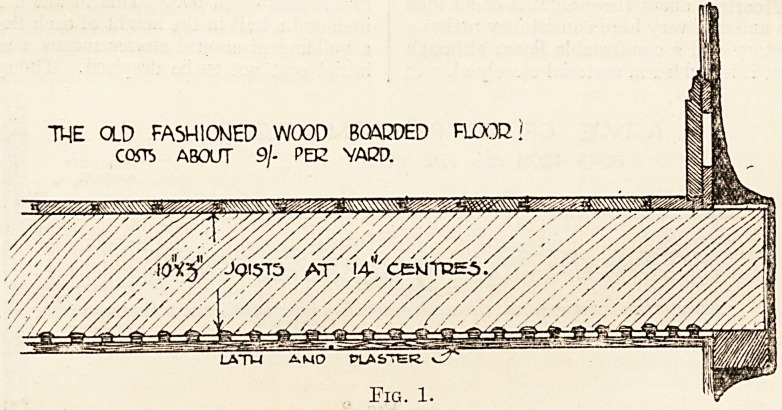


**Fig. 2. f2:**
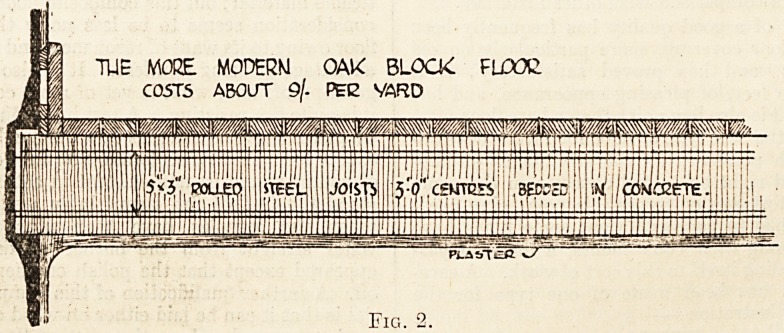


**Fig. 3. f3:**